# Perceptions and experiences of allopathic health practitioners on collaboration with traditional health practitioners in post-apartheid South Africa

**DOI:** 10.4102/phcfm.v8i2.1007

**Published:** 2016-06-10

**Authors:** Simon M. Nemutandani, Stephen J. Hendricks, Mavis F. Mulaudzi

**Affiliations:** 1 Public Health Department, School of Health Sciences, University of Limpopo, South Africa; 2 School of Health System and Public Health, University of Pretoria, South Africa; 3 Nursing Department, University of Pretoria, South Africa

## Abstract

**Background:**

The indigenous health system was perceived to be a threat to the allopathic health system. It was associated with ‘witchcraft’, and actively discouraged, and repressed through prohibition laws. The introduction of the *Traditional Health Practitioners Act* No 22 of 2007 brought hope that those centuries of disrespect for traditional health systems would change. The study examined the perceptions and experiences of allopathic health practitioners on collaboration with traditional health practitioners in post-apartheid South Africa.

**Methods:**

Qualitative descriptive research methodology was used to collect data from allopathic health practitioners employed by Limpopo’s Department of Health. In-depth focus group discussions and meetings were conducted between January and August 2014. Perceptions and experiences of working with traditional health practitioners were explored. Ethical clearance was obtained from the University of Pretoria and approval from the Department’s Research Committee.

**Results:**

Dominant views were that the two health systems were not compatible with respect to the science involved and the source of knowledge. Overall, quality of health care will be compromised if traditional health practitioners are allowed to work in public health facilities.

**Conclusion:**

Allopathic health practitioners do not appear ready to work with traditional health practitioners, citing challenges of quality of health care, differences regarding concept of sciences and source of knowledge; and lack of policy on collaboration. Lack of exposure to traditional medicine seems to impede opportunities to accept and work with traditional healers. Exposure and training at undergraduate level regarding the traditional health system is recommended. Policy guidelines on collaborations are urgently required.

## Introduction

The abolition of the *Witchcraft Suppression Act* of 1957 and the promulgation of the *Traditional Health Practitioners Act* (No 22 of 2007) marked an important epoch in the history of the new democratic South Africa. It symbolised the respect and recognition of traditional health practitioners as forming part of key stake holders in the provision of health services.^1^ It is regarded as an initial milestone in the development of indigenous health knowledge and strengthening the existing interaction between traditional health practitioners and the incorporation of traditional health practitioners in biomedical health-care system.^2,3,4,5^ It further highlighted the importance of acknowledging African heritage and need for change to embrace the existing cultural diversities, community health practices and belief systems in the South African health-care system.

Changing the existing perception towards traditional practices and developing them parallel to the Western practices may require a process of decolonisation of mindset and change of attitudes. In 1978, the World Health Organization’s ‘Alma-Ata’ conference called for official recognition of traditional health practitioners and their integration into national health systems, particularly at the level of primary health care.^6^ About half of the population of African has no access to allopathic health system.^7,8^ Access barriers included vast distances and high travel costs, especially in rural areas; high out-of-pocket payments for care;^9^ long queues;^10^ poor working conditions and shortages of health professionals.^11^

For years, the African traditional health system was perceived to be a threat to allopathic health system’s monopoly over patients’ health and Western religious beliefs.^12,13^ This threat created tensions to which a large extent opposed the recognition and acceptance of traditional health practitioners into the main health system. Summerton suggested that the divergent views on the ‘science of diseases’ contributed to the problem: allopathic medicine looks at ‘material causation’ to understand and treat an illness, while traditional medicine looks towards the ‘spiritual’ origins such as cosmic powers and displeasure by ancestors in order to cure an ailment.^13^

Before 1994, traditional medicine and its beliefs were outlawed in South Africa.^14^ It associated traditional health systems and culture with ‘witchcraft’. The apartheid government actively discouraged and often repressed through violence and other means of prohibition and coercion.^14,15,16^ Despite their suppression and the structural arrangements which ignored traditional medicine and promoted the dominance of allopathic health-care system as the preferred health providers, patients continued to refer themselves to traditional health practitioners.

From the early years of the transition from apartheid to democracy, researchers have been debating on the role of traditional health practitioners.^17,18,19,20,21,22^ It is estimated that there are between 300 000 and 493 000 traditional health practitioners in South Africa.^23^ They constitute a valuable team of community health workers dedicated to providing inexpensive primary health-care services. For instances, both the diagnosed and undiagnosed AIDS patients continue to consult traditional health practitioners-seeking physical, emotional and spiritual relief from AIDS.^24,25^ They are readily available and mostly accessible to the vast majority of communities in sub-Saharan Africa.^26,27,28^

This strong belief on the traditional health system and the dual consultation by the indigenous communities highlights some of the complex processes of seeking treatment in South Africa.^16,19,29^ Recent reports^24^ indicate that South Africa is struggling to achieve Millennium Development Goals 4, 5 and 6. With the current staff shortages of health professionals, and 6 million people with HIV and AIDS, of whom 4 million are believed to consult traditional health practitioners, traditional health practitioners could play potential roles as community health educators, home-based carers and patients supporters.^26^ It is hoped that by working with them, Millennium Developmental Goals 4, 5 and 6 could be achieved.

A number of African countries tried to integrate traditional health systems into their mainstream health system with little success. It has been characterised as one-sided referrals – from traditional health practitioners to allopathic systems.^16,27^ In Zambia, Kaboru and others^30^ found that 40% of allopathic health practitioners expressed interest in working with traditional health practitioners. Obstacles identified by AHPs for collaboration with THPs were lack of any policy, as well as scepticism regarding the science and quality of health care they provide. Additionally, the types of collaboration consisted of AHPs training THPs, rather than the two groups learning together and from each other.

In South Africa, health authorities have accepted the existence and institutions of traditional health practitioners. However, this acceptance seems to be based on terms and conditions acceptable to allopathic medicine, allowing traditional health practitioners to coexist in a pluralism of health-care systems, rather than to incorporate them in the official national health-care system.^16,23^

To date, and despite reports indicating that traditional health practitioners play a vital role in the country’s health system and that they are the patients’ preferred health providers,^7,16,29^ the WHO’s proposal seems to largely have ‘fallen on deaf ears’ of health authorities in Africa.^18,23^ Although public health services in South Africa are free, the latest study report indicated that a higher proportion of people residing in Limpopo failed to consult a health worker when ill or injured due to transportation challenges and staff attitudes. Self-referral to traditional health practitioners has been reported to be common practices among African communities.^31^

The formal recognition of traditional sciences and its integration into allopathic health-care services has been controversial for some time. Many arguments have been offered for and against their incorporation.^16,27,30^ Despite the strength of modern medicine, it is the patients’ belief and attitude that will always determine the type of health care they opt for. Patients, especially women with ‘goni’ (vaginal infection) usually sneak from the hospital to go and consult with THPs.^32,33^

The current study assessed the perceptions and experiences of allopathic health practitioners on collaboration with traditional health practitioners in the new democratic South Africa.

## Methodology

A qualitative, descriptive research method was used to collect data between January and August 2014. Qualitative research methods, in relation to quantitative methods, are flexible in that they allow the researcher to develop concepts during data collection to develop new ideas. This ensures continual interaction between data and existing ideas during data collection.

### Setting

The study was conducted in Vhembe District, which is one of the 11 National Health Insurance’s (NHI) Pilot Districts. The NHI plan was established to ensure that everyone in South Africa has access to appropriate, efficient and quality health services.^34^ The Vhembe District health services consist of 6 district hospitals, 112 primary health-care clinics, 8 community health centres and 407 mobile clinics. There is also one regional hospital as well as a specialised psychiatry hospital situated in the district. It has a population of approximately 1.3 million people.^35^ The data presented here were part of a bigger study to develop a model of collaboration between traditional and allopathic health practitioners in the management of HIV and/or AIDS and TB patients in Vhembe District, Limpopo Province, South Africa.

### Study population

The study population comprised professional nurses, clinical psychologists, social workers, pharmacists, dieticians, medical doctors and/or clinical managers, HIV and/or AIDS and TB programme managers working under public health facilities in Vhembe District, Limpopo Province. The above categories form a team that manage and interact with HIV and/or AIDS and TB patients regularly. Added to that was the district health management team in Vhembe District and the Senior Health Managers in the Limpopo Department of Health. They implement and evaluate health policies and regulations.

### Sampling and sample size

Purposive sampling was used in order to obtain the broadest range of information, perspectives and experiences with regard to working with traditional health practitioners in the fight against HIV and/or AIDS and TB. Three participant characteristics guided sampling strategy. Firstly, as we are interested in obtaining perceptions and experiences of AHPs with regard to working with THPs in the fight against HIV and/or AIDS and TB diseases, we purposively sampled participants who were working at HIV and/or AIDS clinics. They should have 2 years working experience at the wellness clinics. The sample size was reached after there was saturation of the ideas, which were under discussion.

Secondly, we included members of a district management team – including hospital chief executive officers and senior managers at the district level. They are overall managers responsible for health services in the district and play an important role in the evaluation and implementation of health policies. The sample size was 23 participants.

Thirdly, although not directly working in Vhembe District, senior health managers in the Limpopo Department of Health were included due to the fact that they develop policies for implementation at district levels. The researchers were interested in their views with regard to integrating THPs in the main health system and whether there were any policy issues which require attention. The sample size of this study was 10 senior health managers.

### Ethical considerations

The Faculty of Health’s Research and Ethics Committee of the University of Pretoria (REC: 399/2013) and the Limpopo Department of Health (PMREC-54/2013) provided approval. Formal informed consent from participants was acquired and signed on the day of the interview.

### Data collection

Data collection instruments were focus group discussions with AHPs who were working at wellness clinics. Semi-structured interviews were used to collect data during the consultative meetings at Vhembe District and Provincial Health, the number of participants in the focus group discussions ranged from 13 to 18. Focus group sessions were conducted at the health facility’s boardroom and lasted approximately 55 min. Perceptions and experiences of working with traditional health practitioners were explored during focus group discussions.

Each focus group discussion was led by the main researcher (MSN). The use of audiotapes to aid in data collection was explained and accepted by participants. As part of an introduction and to ‘break the ice’, a self-introduction of each member of the group, including years of experiences and their profession, was carried out. This was followed by a statement of the overall purpose of the group discussion and a review of the ground rules during the session. Participants preferred to use English during the meeting. At the beginning of the interview, basic information on the awareness and the knowledge of the *Traditional Health Practitioners Act* no 22 of 2007 was collected. Participants were asked to reflect on: (a) what comes to mind when they hear about traditional health practitioners being accepted and recognised as health providers; (b) their experiences with regard to working with traditional health practitioners and conditions of the patients referred to traditional health practitioners; and (c) to define the nature, type and terms of conditions under which they would consider working with traditional health practitioners.

The role of the main researcher was to guide the discussion to remain focused on the central research question of exploring whether allopathic health practitioners, through their perceptions and experiences, were ready to work with traditional health practitioners in post-apartheid South Africa. Participants were encouraged to express their views and recount personal experiences with patients and traditional health practitioners. An experienced research assistant, Patrick Matumba (PM), trained by Statistics SA, was there to note any non-verbal actions ranging from facial expressions, body posturing, nodding and shaking of heads, and eye movement. To maintain confidentiality and the protection of the participants, their statements were not linked to individual participants. Pseudo names and types of allopathic health providers were linked. At the end of the session, the main researcher summarised the key points discussed and checked with the participants if there were other issues related to the study which would require further clarification.

### Consultative meetings

After an introduction of the members, the main researcher gave an overall purpose of the consultative meeting by means of a 5–8 min PowerPoint presentation. The purpose of the consultative meeting was to brief them about the study project as well as assess their knowledge of the *Traditional Health Practitioners Act* no 22 of 2007 and opinion on integration with THPs. Their comments, views and suggestions regarding nature of collaboration were captured and noted by the main researcher and research assistant during the meeting.

After the meeting, notes were compared and consolidated. Consolidated notes were further checked with views from the Head of Health Branch for trustworthiness – this is the term used to describe the rigour and validity of qualitative research.^34^

### Data analysis

A qualitative data analysis technique, Glaser’s approach of grounded theory was applied.^34^ Copies of the original audiotapes were transcribed by the research assistant. The focus group transcripts were then checked for accuracy and verified against the original audiotapes by the main researcher. Emerging themes, independent meaningful units and segment of text, which were relevant to the purpose of the study, were identified, labelled and organised by the main researcher, in a process referred to as coding.^35,36^ After the initial coding was completed, hand written notes from the two consultative meetings were brought in for the data to be jointly reviewed, reinterpreted, and reorganised into categories with a clinical psychologist and pharmacist. This iterative process was used to search for common patterns and themes, contrast and irregularities; and systematic relationships, among the codes and categories. The final interpretation of the data and conceptual categories were reviewed with the research assistant (PM) and one of the focus group participants.

The trustworthiness of this study was ensured by collecting data from multiple and different sources of information. The triangulated data sources were assessed against one another to cross-check data and interpretation with a clinical psychologist and a pharmacist. The underlying assumption was that the various methods applied and the data collected will complement each other and their respective shortcomings can be balanced out through triangulation process.^36^

## Results

### Focus group discussions

The study achieved saturation with six focus group discussions. Table 1 presents the categories of the participants. A total of 77 participants were interviewed: 38 professional nurses; 12 medical doctors; 6 pharmacists; 6 HIV and/or AIDS program managers; 5 clinical psychologists; 5 dieticians; 3 social workers and 2 clinical managers.

**TABLE 1:** Categories of the participants in the focus groups interviews.

Focus groups	Professional nurse *n* (%)	Doctor *n* (%)	Pharmacist *n* (%)	HIV co-ordinator manager *n* (%)	Clinical psychologist *n* (%)	Dietician *n* (%)	Social worker *n* (%)	Clinical manager *n* (%)
Tshilidzini Hospital	7	3	1	1	1	1	1	-
Silloam Hospital	6	2	1	1	1	1	-	1
Malamulele Hospital	7	2	1	1	1	1	-	1
Tshilwavhusiku Health Centre	6	-	-	1	-	–	1	-
Elim Hospital	4	4	2	1	1	1	1	-
Donald Frazer Hospital	8	1	1	1	1	1	-	-

**Total (male)**	**38 (23)**	**12 (8)**	**6 (5)**	**6 (6)**	**5 (2)**	**5 (1)**	**3 (2)**	**2 (2)**

*Source*: Authors’ own work

### Consultative meetings with management

Table 2 presents the characteristics of participants who attended the consultative meetings at the district and provincial levels.

**TABLE 2:** Provincial health branch management team.

Provincial health information	Gender	Experience in years	Number	Average working experience
**Job title**				
Senior general manager: Health	Male	20	-	-
General manager: HIV	Male	12	-	-
General manager: Strategic	Male	8	-	-
General manager: PHC	Male	9	-	-
General manager: Hospital	Male	25	-	-
General manager: Vhembe	Male	6	-	-
General manager: Mopani	Male	7	-	-
General manager: Sekhukhune	Female	7	-	-
General manager: Capricorn	Male	6	-	-
General manager: Waterberg	Female	4	-	-
**District health management team**				
Sub-district HIV co-ordinators	-	-	11	4 years
Hospital CEOs	-	-	7	4 years
Senior managers	-	-	3	2 years
Managers	-	-	2	3 years

*Source*: Authors’ own work

Most of the positions at the provincial health management team were occupied by males. The average working experience was around 7 years.

A total of 23 out of 36 members of the district management team attended the semi-structured interview meeting. The majority of the members were sub-district HIV and/ or AIDS co-ordinators (11), followed by hospital CEOs (7) and senior managers at the district level (3). Most participants had 4 years working experience in the public sector.

Health managers at both the district and provincial levels seemed to be more welcoming and open to the idea of integrating THPs into their health system. The initial remark by Head of Health Branch, during the introduction to the senior health managers, summarised it all in the following statement:

‘… It is long overdue that we start to recognise that there are others [*THPs*] who renders quality health services to thousands if not millions of our people. It’s unfortunate that their sciences and knowledge of medicine, plants and animals has not been acknowledged or documented for us. The Traditional Act has been developed for too long, it must be put into action. We hope to hear from you, how you plan to do that …’ (Head of Health Branch – 0)

With the exception of one district executive manager, all members of health branch were aware of the existence of the Act. At both levels (district and provincial), the knowledge of the Act contents, especially with regard to the definition of THPs, scope of practice and their recognition as health professionals, and the need for integration with other health providers such as allopathic health practitioners appeared vague. There was general consensus in all the meetings about the need to work together with THPs. However, concern was raised with regard to their standard of care; patient safety; medicine storage; overdose; false promises, especially that of a cure for HIV and/or AIDS; and unscrupulous practices and the lack of control measures. These concerns were clearly demonstrated by the following comments:

‘… what do they say about their claim to cure HIV/AIDS … our patients will not be safe.’ (CEO – 1)

‘I have once visited their clinics, everything is mixed up, no order at all … you know … Herbs and skin of animals hanging on the walls.’ (HIV co-ordinator – 3)

### Themes and subthemes

Analyses of participants’ narratives and consultative discussions resulted in three main themes: (1) quality of health care, (2) concept of sciences and source of knowledge, (3) policy guidelines and training (Table 3). The themes are introduced first; then subthemes are substantiated by direct quotations from participants.

**TABLE 3:** Themes and subthemes.

Theme	Subtheme
Quality of health care	Lack of HIV and/or AIDS knowledge, skills and resources. Traditional health practitioners interfered with the efficacy of antiretroviral treatment.
Concept of sciences and source of knowledge	Traditional medicine uses unscientific methods and standards of care. It operates under secrecy and superstitions.
Policy guidelines and training	Support for the development of policy on collaboration and training workshops on HIV and/or AIDS and TB.

*Source*: Authors’ work

### Theme 1: Quality of health care

The first theme that emerged from the group discussions was on the quality of health care which AHPs strongly believe that it was being compromised by recognising THPs as health professionals alongside AHPs. There was generally a lack of knowledge among allopathic health practitioners on the Act regulating traditional health practitioners’ matters in South Africa. Some of the participants were not even aware that traditional health practitioners are to be recognised as health professionals and they should be integrated into the main health system:

‘Does it mean that they [*THPs*] are now health professionals like us? What is going on now? But we were never consulted about this Act … How is it going to work?’ (Clinical manager – 3)

‘I know these politicians, they just approve without considering the consequences of their actions. Ahh ! They don’t care …’ (Clinical psychologist – 1)

The concern about the quality of health care was further divided into two subthemes. The first subtheme was THPs lack of knowledge, skills and resources to manage HIV and/or AIDS and TB patients. The second subtheme was that they interfered with the efficacy of ARV and TB treatment.

Concern was raised about the Act and their view was that allowing and recognising them to work with allopathic health practitioners would cause more harm for the patients and affect quality of health care being rendered in public health facilities. The following statements highlight some of the concerns raised during the discussion:

‘… What I see here is that there will be more death now, if we go to medical wards, there are lots of patients with renal failures, patients come here at critical stages, we cannot resuscitate them. Relatives take our patients on treatment to the THPs, only to return them when they are critical …’ (Medical doctor – 2)

‘…’The truth is, when the patients are critically ill, they [*relatives*] take them to the hospital, and not to traditional health practitioners. So, really the death rate will be very high. And the hospitals would look like they are not working …’ (Clinical psychologist – 4)

‘… Oh … that is the end of it. Imagine them coming to work in our hospital, throwing bones and beating drum next to you. Our standards are being compromised …’ (Social worker – 3)

Although the attitudes and views expressed by participants differed from one individual to another, they all acknowledged that THPs were: … silently stealing our patients with the false promise and hope of cure for HIV/AIDS and other diseases. A doctor narrated a story of what happened:

… they took ARV tablets, crash them into powder and mix it with their herbs and claim that they cure HIV and/or AIDS …’ (Pharmacist – 1)

This was supported by professional nurse as follows:

‘… I am telling you, this will not work. Government should consult widely when they make these laws. Our value system, science of medicine and standard of care will be compromised if we recognise and accept traditional healers to treat our patients …’ (Professional nurse – 5)

### Theme 2: Concept of sciences and source of knowledge

The second theme explored the concept of sciences and source of knowledge, which they strongly believed was not comparable and compatible to science. This theme developed from two subthemes: The first subtheme being that traditional medicine is unscientific. It is based on superstitions, secrecy and spirits. The other subtheme being that of secrecy and lack of scientific method.

A social worker supported by a pharmacist insisted that:

‘… Traditional health practitioners believe in ritual killings, are we not supporting them if we work with them? They believe that illness is caused by witches, we don’t believe in witches. How can the two work together? We first have to get same understanding, concept of diseases and sciences involved …’ (Social worker – 1)

Participants felt that this difference in understanding of the cause of illness will result in constant conflict and an unworkable environment. Source of THPs knowledge, which is ancestors, will not allow them to learn from allopathic health practitioners. For now, they would prefer that THPs continue to refer patients to allopathic health practitioners. On probing further, participants felt that they should train THPs on Western medicine. Trust and credibility were key issues in developing working relationships. There was no doubt among the participants that their medicine works. One of the participants expressed the view that:

‘… It’s only that we don’t know how it works, and they are not prepared to let us know what it is. Their medicine is very powerful and toxic, so much that when they come with toxicity, renal failures are eminent …’ (Clinical manager – 3)

### Theme 3: Policy guidelines and training

Beyond the existing differences in sciences and quality of care stated, participants felt that working with THPs would require a good explanation, in a form of circular and/or policy directives from provincial office to the districts, providing details on how the two should work together. It was also the main subtheme, suggesting that THPs should be workshopped on HIV and/or AIDS and TB diseases:

‘… If they are properly trained, we will work with them as ARV and DOT supporters. Will they not mix our medicine with their herbs …? (Medical doctor – 1)

They felt that more training on signs and symptoms of HIV and/or AIDS and TB should be conducted among THPs. This would make it easy for THPs to refer patients to them. Referrals from AHPs to them were not supported.

## Discussion

The dominant view was that working with THPs would not work, it will compromise quality and standard of health care provided, increase death rate and delay patients from seeking treatment and consulting them. One gets the impression that participants do not support the Act, as a result of subjective fear for THPs taking away and treating their patients with ‘untested’ herbal medicines, which would result more death, drug toxicity, renal failure and drug resistance.

In recent years, the South African National Department of Health (NDoH) has shown an unwavering commitment to improving the quality of health care. This commitment was further cast into the spotlight through the publication of the 10 Point Plan for improvement of the health sector (2012–2014) in July 2010 and the introduction of an NHI plan.^2,34^ Quality in health care refers to the extent to which an organisation meets its clients’ needs and expectations.^37^ It should address patient’s needs which range from physical, mental and psychological state of mind among others. It is the patients unmet needs which calls for the two to work together. Abdool Karim et al.^37^ argued that the reluctance on the part of AHPs to collaborate, is a result of them not accepting that patients have a right to consult THPs. Despite the strength of modern medicine, clients’ and patients’ beliefs and attitudes will always determine the type of health care they seek.^37^

Contrary to our participants’ views, a controlled study of an HIV/AIDS/STI/TB intervention with THPs in SA found that working with THPs had more benefits for the patients. They reported a high level of commitment in the fight against HIV and/or AIDS and TB.^26,38^ In 2006, Summerton suggested that by using the strength of the traditional healing system, ‘treating patients psychologically, and scientifically unexplained physiological relief of the symptoms of specific illnesses’, the inefficient Western health-care system would be benefitting more.^13^

Various factors impede effective collaboration between traditional and allopathic health practitioners in South Africa. King and others attribute the difficulty in integrating traditional and allopathic health practitioners to the prejudicial notion among allopathic health practitioners, that traditional African beliefs and practices have no scientific basis, they are ‘primitive’ and ‘savage’, and ‘witches’ practicing black magic.^28^ Peltzer^27,38^ reiterated this notion by pointing out that Western health practitioners’ critical view of traditional medicine is based on notions which perceive traditional health practitioners as posing a danger to the health of their patients, as also reported by our participants.

Participants described that in their views, the two health systems have a different understanding of the sciences. Their perception was that THPs would not be able to systematically investigate diseases, assess the patient and provide diagnosis. This would make it very difficult for AHPs to work with them, let alone to refer patients to them. Added to that, was the fact that THPs are associated with witchcraft and evil powers.

The general consensus was that the two health systems do not trust each other as a result of their different sciences and sources of knowledge. The difference in understanding sciences and source of knowledge has been controversial for some time. According to *Webster’s New Collegiate Dictionary*, science is an ‘organized body of knowledge attained through study or practice’. Unlike allopathic science which is acquired through study and/or formal learning, traditional sciences or knowledge is passed from ancestors, who are the custodians of culture and source of knowledge practiced by traditional health practitioners, to human beings irrespective of age and capacity to comprehend or study. As a result of these different viewpoints, many arguments have been offered for and against their incorporation. Sindiga^39^ argued that part of the misunderstanding regarding African traditional healers emanates from a historical ‘blind spot’ of colonisation and westernisation by missionaries.^3^ Historically, and views also expressed by our participants, traditional health practitioners were dubbed ‘witches’ practising black magic. Abdool Karim and others^37^ argued that this ‘blind spot’ developed over years, has been preventing allopathic health practitioners from working with THPs.

The scepticism with which AHPs view traditional healing and practitioners thereof is not wholly unjustified. Various factors contribute towards such scepticism, including the lack of knowledge among AHPs about traditional theories of disease and health.^39^ In most cases, it is the mysticism surrounding traditional medicines (content), the harmful traditional healing practices, herbal intoxications and patients abandoning HIV and/or AIDS and TB treatment for herbal medications. However, these negative factors associated with the traditional health care system might not justify the exclusion of traditional health practitioners from the realm of health care in general,^40^ and priority programmes such as HIV and/or AIDS. A significant percentage (60% – 80%) of patients consult them.^38^ In terms of the HIV and/or AIDS pandemic, it would mean that between 60% and 80% (4–5 million HIV+ patients) of the reported 6 million South African diagnosed HIV patients, who are consulting THPs today, could be lost in-between the two health systems.

As AHPs were not aware of the existence of the Act, it is not surprising that they would want detailed information at district level on how to apply the Act in their work environment through departmental circulars or policy. So far, there is no policy on how collaboration should be structured, implemented and monitored. If policy was developed, the implementation of it through the series of consequential steps, interaction and negotiations with all stakeholders would be less resisted. A top-down approach is more useful when goals and objective are clearer and policies are designed in a comprehensive way. We would not be recommending a top-down approach, as our participants already expressed their disapproval of the Act. Local experiences and perspective are important factors, which contribute to success or failure of any public policy. We are in favour of a bottom-up approach. It is helpful for implementation if objectives are clear and polices are understood. Our participants were not clear about the content of the Act.

## Conclusion

Based on the findings of the study, some salient conclusions have been drawn which can inform policy. In analysing the perceptions of AHPs, we concluded that they were concerned that collaboration with THPs would compromise patients’ health care. Until AHPs are convinced that the THPs’ sciences and knowledge of medicine play a major role among patients and communities in South Africa, they will continue to distance themselves from cooperating with THPs. Despite the fact that ‘their patients’ were also consulting traditional health practitioners, they maintained the view that collaboration would not be in the best interest of patients, and thus, they were not ready to work with them. Lack of existing policy on how and under what conditions they should collaborate and their limited exposure to the nature of traditional healers’ scope of practices and sciences involved seem to impede opportunities to accept and work with traditional healers.

### Recommendations

The following recommendations are presented based on the findings and conclusions of the study:

1. Exposure to traditional practices and their sciences at undergraduate level of study is the corner stone in developing trust and exchanging knowledge. Universities should consider introducing a module on traditional medicine, and should be encouraged to explore and expand their community-based learning to include traditional health practitioners.
2. Consultative process and open dialogue between the two health systems should be encouraged.
3. The Department of Health at national level should develop policy guidelines on how integration with traditional health practitioners should be implemented.

### Limitations

The study is limited to the context of perceptions and experiences of AHPs in Vhembe District. Purposive sampling was the other limitation for this study.

Self-reported data can rarely be independently verified and may contain several potential sources of bias such as: (1) selective memory for remembering or not remembering experiences or events that occurred at some point in the past; (2) attribution – the act of attributing positive events and outcomes to one’s own agency but attributing negative events and outcomes to external forces; and, (3) exaggeration – the act of representing outcomes or events as more significant than is actually suggested from other data.
